# Support for Care Economy Policies by Political Affiliation and Caregiving Responsibilities

**DOI:** 10.1001/jamahealthforum.2025.1204

**Published:** 2025-06-06

**Authors:** Katherine E. M. Miller, Jennifer L. Wolff, Karen Shen, Sandro Galea, Catherine K. Ettman

**Affiliations:** 1 Department of Health Policy and Management, Bloomberg School of Public Health, Johns Hopkins University, Baltimore, Maryland; 2 Office of the Dean, Boston University School of Public Health, Boston, Massachusetts; 3 Editor, *JAMA Health Forum*

## Abstract

**Importance:**

Identifying effective and financially viable strategies to meet the care needs of perons with impaired function is a policy challenge for high-income countries with aging populations. The 2022 National Strategy to Support Family Caregivers identified a range of actions to support caregivers, while family-oriented policies to promote the affordability of care were promoted by both candidates in the 2024 presidential election.

**Objective:**

To examine public perceptions of federal policies to support older adults, adults living with disabilities, and their family caregivers by political affiliation and caregiving status.

**Design, Setting, and Participants:**

This cohort study included adults in the CLIMB study, a nationally representative, longitudinal panel. Data were collected in March and April 2024. Data analyses occurred from September 2024 to February 2025.

**Exposures:**

Self-reported political party affiliation and caregiving responsibility.

**Main Outcomes and Measures:**

Endorsement of 6 policies. Responses based on a 5-point Likert scale were dichotomized to contrast “strongly” or “somewhat support” with “strongly oppose,” “somewhat oppose,” and “neither support nor oppose.” The probability of support and differences across political affiliations was presented.

**Results:**

Of 2059 respondents (1035 female individuals [50.9%]; mean [SD] age, 49.0 [18.2] years), 394 (20%) reported having caregiving responsibilities, with no significant difference across political affiliation. Endorsement was highest for policies to make care in facilities (1657 [79.0%]) and homes (1600 [75.4%]) more affordable, expand eligibility for financial access to care (1618 [77.3%]), and increase the capacity of the paid caregiving workforce (1649 [78.3%]) and was lower for expansion of paid family leave (1342 [65.4%]) and payment of family caregivers (1223 [61.2%]). Endorsement by political affiliation was most similar for policies to make care at home more affordable (13.7–percentage point difference; 95% CI, −20.4 to −7.1) and least similar for paid family leave (33.4–percentage point difference; 95% CI, −39.0 to −27.7). While respondents with caregiving responsibilities were more likely to support paying family caregivers, political affiliation was associated with the endorsement of policies to support the care economy that was stronger in magnitude than sociodemographic characteristics (eg, sex) or caregiving experiences.

**Conclusions and Relevance:**

The results of this cohort study suggest that, despite some differences by political affiliation, there is high support of policies to support the adult care economy, suggesting a policy window to advance legislation and executive action to address the care needs of aging populations and populations with disabilities.

## Key Points

**Question:**

Does public opinion on policies supporting the care economy differ by political affiliation or caregiving responsibilities?

**Findings:**

In this cohort study of 2059 individuals, one-fifth of US adults reported having caregiving responsibilities for an adult with a disability, regardless of political affiliation, and there was widespread support for policies to sustain the care economy across demographic characteristics, caregiver status, and political affiliation. The policies most highly endorsed by Democrats and Republicans were those addressing affordability.

**Meaning:**

The study results suggest that public support of the care economy is strong across caregivers and noncaregivers as well as political affiliation and that policy action is a promising priority with bipartisan support.

## Teaser

This cohort study examines public perceptions of federal policies to support older adults, adults living with disabilities, and their family caregivers by political affiliation and caregiving status.

## Introduction

Advancing affordable, dignified, person-directed care is a critical longstanding national priority^1,2,3,4^ that has become more evident with population aging.^5,6,7^ The US lacks a holistic approach to meeting the care needs of those living with disabilities. In the absence of a long-term care insurance system, most costs are borne out of pocket by individuals and families or by Medicaid, a program for persons with low incomes and assets.^8,9,10,11^ Most care needs are met by family (unpaid) caregivers who provide hands-on help with personal care, medical tasks, and household chores,^12,13,14,15,16,17^ with little or no training.^18,19,20,21,22^ For the approximately half of family caregivers who are employed, caregiving responsibilities may be associated with reduced labor force participation and work productivity.^23,24,25,26,27,28,29,30,31,32,33,34,35,36^ Paid caregivers are an important source of supplemental help and respite, but in the absence of insurance and in light of workforce recruitment and retention challenges, this care is expensive and difficult to access.^37,38^

Many factors have reinvigorated a national policy debate on the care economy, ranging from discussions of value-based payment policies to a growing awareness of challenges in accessing affordable care as the population ages. The Administration for Community Living, within the US Department of Health and Human Services, released the first National Strategy to Support Family Caregivers in 2022.^39^ President Biden’s April 2023 executive order (#14095) called for greater access to and affordability of high-quality care for adults living with cognitive and/or functional disabilities by improving working conditions for paid caregivers.^40^ Without a cohesive federal strategy, states have been at the forefront in advancing home and community-based supports, including expansions of services offered by Medicaid and enacting paid family leave programs.^41^ Additionally, with a politically divided populace,^42^ having support across political parties will be key for the passage and success of proposed policy solutions.

Expanded coverage of home care within Medicare was proposed during the 2024 election as a policy priority,^43,44,45^ yet how these policies are viewed across partisan lines is unclear, as such information is not routinely collected in ongoing national surveys. Family caregivers generally endorse financial support and investments in the long-term care system,^39,46,47^ yet comparative information about noncaregivers or variation in political party affiliation is not currently known. This study drew on a recent nationally representative survey of adults in the US to describe the endorsement of policies proposed in the National Strategy to Support Family Caregivers, contrasting endorsement by political affiliation and caregiving responsibilities with the goal of informing national conversations and policy.

## Methods

### Study Design, Population, and Setting

This was a cross-sectional study of a nationally representative survey of adults 18 years or older in the US. We followed the Strengthening the Reporting of Observational Studies in Epidemiology (STROBE) reporting guidelines.^48^ We used data from the fifth wave of the longitudinal CLIMB study, which were collected in March and April 2024.^49,50,51,52,53,54^ The National Opinion Research Center recruits respondents to CLIMB from the AmeriSpeak panel, a probability-based panel covering 97% of US households. All AmeriSpeak participants provided informed consent before starting the CLIMB survey. If participants completed the survey online, then written consent was obtained. If participants completed the survey via phone, then oral consent was obtained. In the fifth wave, the CLIMB survey instrument included a panel of new questions regarding (1) past, current, and expected future status as a family caregiver and (2) endorsement of policies that aligned with the National Strategy to Support Family Caregivers, a contribution to existing datasets used to study family caregiving. Of the 3005 adults invited to participate, 2144 (71.3%) responded in 2024. We excluded respondents with missing values from our analysis (n = 85), yielding an analytic sample of 2059. This study was found to be exempt by the institutional review board of National Opinion Research Center at the University of Chicago for original data collection and was deemed not human participants research for deidentified secondary data analysis by Johns Hopkins University and Boston University Medical Center.

### Measures

Outcomes included the endorsement of 6 policies aligned with (1) actions outlined in the National Strategy to Support Family Caregivers and (2) ongoing policy discussions (eg, President Biden’s executive order addressing access to affordable care and investments in care workers). For each policy, respondents were asked “The federal government is considering a plan for family and other unpaid caregivers who help children and adults for health and functioning needs. Some of the policies that have been proposed are listed below. They would each cost money. On a scale of 1 to 5 where 1 is strongly oppose and 5 is strongly support, how much do you support each policy?” We dichotomized responses of 4 (somewhat support) and 5 (strongly support) as 1 and all other nonmissing values as 0, which was consistent with prior work.^55,56^ The 6 policies were categorized within 3 domains. The first doman, making long-term care more affordable and accessible, contained 3 policies: (1), making aging at home more affordable through strengthening the accessibility and quality of services and supports (eg, respite care, adult day care); (2) making long-term care at a facility (eg, nursing home) more affordable; and (3) not requiring people to deplete their assets to be eligible for long-term care. The second domain, expanding the supply and quality of paid care, included a policy to support and expand the professional caregiving workforce. The third domain, relieving the burden of family (unpaid) caregiving, contained 2 policies: (1), providing paid family and medical leave (eg, short-term leave with partial pay) and (2) providing payments to family members or friends who are giving care.

The key explanatory variables of interest were self-reported political affiliation (Democrat, Republican, Independent, or none of these) and caregiving responsibility. We identified current caregivers as individuals who self-reported providing care to a relative or friend 18 years or older to help them take care of themselves (eg, helping with personal needs, household chores, and managing a person’s finances).

Additional explanatory variables included sex, age, race, ethnicity, education, household income, household size, marital status, parental status, metropolitan residence, US Census division, and current caregiver status.^49,55^ Finally, we described employment status among respondents. We included these covariates, as they are associated with long-term services and support use and therefore may be associated with perceptions of support for related policies.^8,57,58,59,60^ See the eMethods in Supplement 1 for survey language regarding outcomes and key explanatory variables.

### Statistical Analysis

First, we calculated the percentage of respondents who reported caregiving responsibilities. Second, we described the sociodemographic characteristics and employment status of the overall sample and by caregiving status, using χ^2^ tests for differences in categorical characteristics and *t* tests for continuous variables across groups. We reported unweighted frequencies and weighted percentages. We then estimated 6 logistic regression models (1 for each policy) to examine the association of political affiliation and endorsement of policies to support the care economy. In all analyses, we used survey weights to adjust for sampling design and nonresponse to yield a nationally representative sample. We also adjusted for age, race, sex, education level, marital status, household income interacted with household size, caregiving status, parental status, metropolitan residence, and US Census division. We calculated the adjusted mean probability of support for each policy, as well as the differential association of each covariate, using the method of recycled predictions. Finally, given the gendered nature of caregiving historically, we conducted sensitivity analyses with gender and political affiliation interacted to identify differential associations of gender by political affiliation. We used Stata SE, version 18.5 (StataCorp). Statistical significance was set at *P *< .05.

## Results

Of the 2059 respondents in the analytic sample, the mean (SD) age was 49.0 (18.1) years; 1035 (50.9%) were female, 315 (16.6%) were Hispanic, 193 (11.6%) were non-Hispanic Black, 1410 (62.4%) were non-Hispanic White, and 141 (9.5%) were another race and non-Hispanic (inclusive of American Indian, Alaskan Native, Asian Indian, Chinese, Filipino, Japanese, Korean, Vietnamese, other Asian, Native Hawaiian, Guamanian or Chamorro, Samoan, other Pacific Islander, and other race) (Table). Respondents indicated Democratic (727 [35.1%]), Republican (521 [25.0%]), Independent (563 [26.5%]) and other (248 [13.6%]) political affiliation.

**Table.** Descriptive Statistics of Cohort by Caregiving Status

Characteristic	No. (%)			*P* value^a^
	Overall sample (N = 2059)	Not a family caregiver (n = 1665)	Family caregiver (n = 394)^b^	
Age, mean (SD), y	49.0 (18.1)	48.6 (18.2)	50.3 (17.7)	.24
Race and ethnicity				
Hispanic	315 (16.6)	250 (16.0)	65 (18.7)	.70
Non-Hispanic Black	193 (11.6)	150 (11.6)	43 (11.7)	
Non-Hispanic White	1410 (62.4)	1150 (62.9)	260 (60.3)	
All other races, non-Hispanic^c^	141 (9.5)	115 (9.5)	26 (9.2)	
Highest level of education				
Less than high school	73 (6.0)	58 (6.4)	15 (4.4)	.64
High school graduate or equivalent	352 (29.8)	283 (29.4)	69 (31.3)	
Some college or associate’s degree	811 (27.7)	649 (27.3)	162 (29.1)	
Bachelor’s degree	457 (20.5)	379 (21.0)	78 (18.5)	
Postgraduate study or professional degree	366 (16.1)	296 (16.0)	70 (16.7)	
Marital status				
Married/living with partner	1302 (59.3)	1062 (59.1)	240 (59.7)	.94
Widowed	110 (5.4)	87 (5.4)	23 (5.6)	
Divorced or separated	276 (12.0)	217 (11.9)	59 (12.4)	
Never married	371 (23.3)	299 (23.6)	72 (22.3)	
Household income, $				
<30 000	357 (20.3)	270 (19.4)	77 (24.3)	.06
30 000 to <50 000	339 (16.2)	254 (15.3)	85 (19.9)	
50 000 to <75 000	432 (20.8)	363 (21.4)	69 (18.3)	
75 000 to <150 000	662 (29.9)	546 (29.8)	116 (30.0)	
≥150 000 or more	269 (12.8)	232 (14.2)	37 (7.2)	
Metropolitan residence				
Nonmetropolitan	307 (15.0)	251 (15.3)	56 (14.0)	.62
Metropolitan	1752 (85.0)	1414 (84.7)	338 (86.0)	
Sex				
Female	1035 (50.9)	803 (48.5)	232 (60.5)	.01
Male	1024 (49.1)	862 (51.5)	162 (39.5)	
Employment status				
Working full time	1002 (45.9)	854 (48.5)	148 (35.4)	<.001
Working part time	216 (10.8)	158 (9.8)	58 (14.9)	
Looking for work or unemployed	76 (5.4)	59 (5.3)	17 (6.0)	
Retired	472 (20.9)	371 (20.8)	101 (21.5)	
Homemaker, student, or other	146 (9.4)	117 (9.3)	29 (9.7)	
On leave (parental, illness/sick, disability)	144 (7.5)	104 (6.3)	40 (12.5)	
Political affiliation				
Democrat	727 (35.1)	589 (35.7)	138 (32.6)	.80
Republican	521 (25.0)	418 (24.6)	103 (26.3)	
Independent	563 (26.4)	459 (26.3)	104 (26.5)	
Other	248 (13.6)	199 (13.4)	49 (14.6)	
Caregiver to child(ren) <18 y	102 (5.4)	0	102 (27.6)	<.001
Endorsement of care economy policies (somewhat or strongly support)				
Making care more affordable/accessible				
Make aging at home more affordable through strengthening the accessibility and quality of services and supports (eg, respite care, adult day)	1600 (75.4)	1284 (75.0)	316 (76.)	.59
Make long-term care at a facility (eg, nursing home) more affordable	1657 (79.0)	1341 (79.5)	316 (76.9)	.43
Do not require people to deplete their assets to be eligible for long-term care	1618 (77.3)	1310 (77.5)	308 (76.1)	.64
Expanding the supply and quality of paid care				
Support and expand the professional caregiving workforce	1649 (78.3)	1321 (77.4)	328 (82.3)	.13
Relieving the burden of family (unpaid) caregiving				
Provide paid family and medical leave (eg, short-term leave with partial pay)	1342 (65.4)	1093 (66.1)	249 (62.6)	.35
Provide payments for family members or friends giving care	1223 (61.2)	971 (59.7)	252 (67.0)	.049

^a^ *t *Test adapted to complex survey samples; χ^2^ test with the second-order correction by Rao and Scott.

^b^ No. (unweighted %).

^c^ American Indian, Alaskan Native, Asian Indian, Chinese, Filipino, Japanese, Korean, Vietnamese, Other Asian, Native Hawaiian, Guamanian or Chamorro, Samoan, Other Pacific Islander, and some other race.

**Figure 1.**
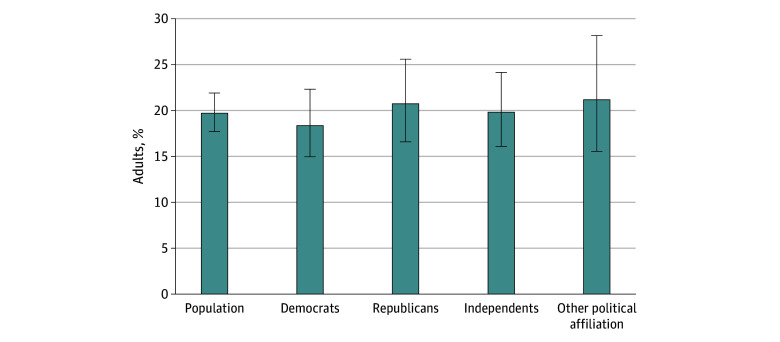
Prevalence of Family Caregiving Nationally and by Political Affiliation Current caregiving status was ascertained from the survey question “At any time in the last 12 months, has anyone in your household provided unpaid care to a relative or friend 18 years or older to help them take care of themselves? This may include helping with personal needs or household chores. It might be managing a person’s finances, arranging for outside services, or visiting regularly to see how they are doing. This adult does need not live with you.” Respondents could respond “Yes, I have provided care to an adult in the last year,” “Yes, someone else in my household has provided care,” or “No.” We defined family caregivers if respondents reported “Yes, I have provided care to an adult in the last year.”

Approximately 20% of US adults reported having caregiving responsibilities for an adult 18 years or older. Caregivers were significantly more likely to be female (232 [60.5%] female vs 162 [48.5%] male) and less likely to be working full time (58 caregivers [14.9%] working part time) than noncaregivers. Among family caregivers of adults, 102 (27.6%) also provided care to a child. Among respondents who were not currently providing caregiving, more than half (855 [51.0%]) indicated an expectation of future caregiving responsibilities. The share that reported caregiving responsibilities was comparable by political affiliation (Figure 1).

**Figure 2.**
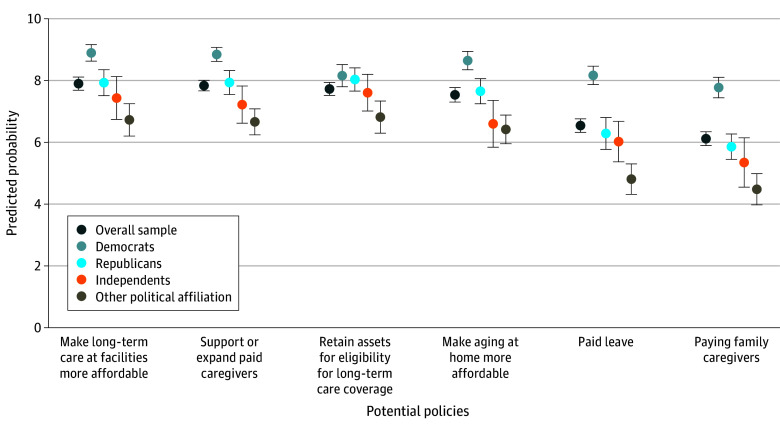
Predicted Probability of Support for Long-Term Care Policies Across Political Affiliations For each policy, respondents were asked “The federal government is considering a plan for family and other unpaid caregivers who help children and adults for health and functioning needs. Some of the policies that have been proposed are listed below. They would each cost money. On a scale of 1 to 5 where 1 is strongly oppose and 5 is strongly support, how much do you support each policy.” We dichotomized responses with strongly ( = 5) or somewhat support ( = 4) as 1 and all other nonmissing values as 0, which was consistent with prior work. We calculated the mean predicted probabilities of supporting each policy type from logistic regression models that adjusted for whether someone is a caregiver, age, race and ethnicity, education, marital status, household income interacted with household size, parent, US Census division, metropolitan residence, political affiliation, and sex. We used sampling weights and specified the primary sampling units and variances to adjust standard errors to account for the complex survey design. The bars around each square reflect the 95% CIs around the mean predicted probability.

We found a high endorsement of policies to promote the affordability, accessibility, and supply of care for persons with disabilities and better support of family caregivers after adjusting for demographic characteristics. Among all respondents, 1657 (79.0%; 95% CI, 76.9%-81.1%) endorsed making long-term care at a facility more affordable, 1649 (78.3%; 95% CI, 76.7%-80.1%) endorsed supporting and expanding the paid caregiving workforce, 1618 (77.3%; 95% CI, 75.2%-79.4%) endorsed not requiring people to deplete assets to be eligible for long-term care coverage, 1600 (75.5%; 95% CI, 73.2%-77.9%) endorsed making aging at home more affordable, 1342 (65.4%; 95% CI, 63.2%-67.6%) endorsed paid family leave, and 1223 (61.2%; 95% CI, 59.0%-63.6%) endorsed paying family caregivers (Figure 2).

Endorsement of policies was highest among Democrats and lowest among Republicans. Nevertheless, most Republicans endorsed 4 of the 6 policies (eTable 1 in Supplement 1 for unadjusted means and Figure 2 for adjusted means). Republicans least often endorsed policies to relieve the economic burden on unpaid family caregivers (48.4% endorsed paid leave and 45.0% endorsed paying family caregivers) and most often endorsed policies to address the affordability of care in facilities (67.5%) and at home (64.1%) and increasing capacity of the paid care workforce (66.6%). Democrats strongly endorsed all 6 policies, ranging from a low of 77.8% for paying family caregivers to a high of 89.0% for making care in facilities more affordable.

**Figure 3.**
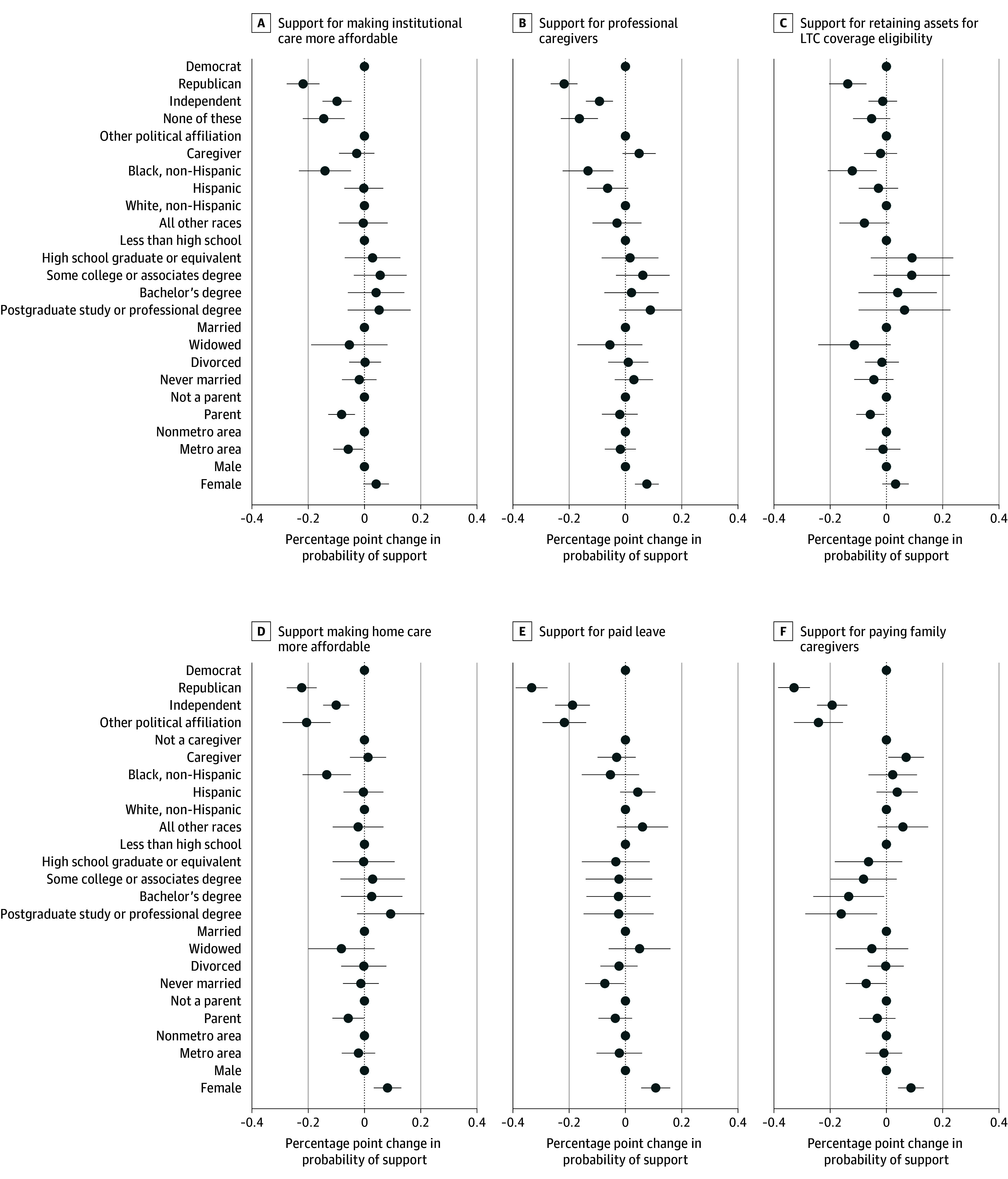
Marginal Effects of Respondent Characteristics and Support for Policies Around the Care Economy We calculated the mean differential effect of supporting each policy type from logistic regression models that adjusted for whether someone is a caregiver, age, race and ethnicity, education, marital status, household income interacted with household size, parent, US Census division, metropolitan residence, political affiliation, and sex. We used sampling weights and specified the primary sampling units and variances to adjust standard errors to account for the complex survey design. The bars around each square reflect the 95% CIs around the mean predicted probability. LTC indicates long-term care; metro, metropolitan.

Political affiliation was the dominant characteristic explaining the endorsement of policies to support adults with disabilities and their families (Figure 3). By contrast, we found few differences between current caregivers and noncaregivers in endorsing policies to support the care economy. Current caregivers were more likely to endorse paying family caregivers than noncaregivers, but endorsement otherwise did not differ by policy. Differences in endorsement of policies by sex, caregiving experience, race, and education were found but were less consistent and smaller in magnitude than political affiliation (Figure 3). Women were consistently more likely to support each policy, while respondents identifying as Black and respondents with higher levels of education were significantly less likely to support policies addressing the affordability of care and investments in the paid care workforce. Differences in the endorsement of policies by political affiliation were the smallest for allowing individuals to retain assets in eligibility criteria for long-term care coverage, with Republicans nearly 13.7 percentage points less likely to support the policy compared with Democrats while holding all covariates in the model constant. The full model results are shown in eTable 2 in Supplement 1.

In sensitivity analyses examining the probability of endorsing each policy, we found no significant effects of sex among Democrats. Among Republicans and those with with affiliation other than Democrat, Independent, or Republican, women were significantly more likely than men to support paid leave and paying family caregivers. Finally, among Independents, women were significantly more likely to support investments in the direct care workforce, paid leave, and making aging in place and care in a facility more affordable. The full model results in shown in eTable 3 in Supplement 1.

## Discussion

In this nationally representative cohort study of US adults, about 1 in 5 US adults reported having current adult caregiving responsibilities across political affiliation. There were high levels of endorsement of policies that supported the care economy, with more than half of respondents expressing support for each of the 6 policies in the survey. Policies to make care more affordable and build the capacity of the paid care workforce were most strongly endorsed. The policy with the most comparable endorsement across political parties was expanded eligibility for long-term care coverage. Policies with the lowest levels of overall endorsement and largest gaps in support by political affiliation were those focused on relieving the burden on unpaid family caregivers (providing paid leave and paying family caregivers), yet these policies were nevertheless endorsed by more than half of all respondents. All but the paid leave and paid family caregiver policies retained majority support across political parties.

Our findings that policies to ensure affordable care are highly endorsed by US adults demonstrate consistency across US adults regardless of caregiving responsibilities^39,46,47^ and may be explained by the affordability crisis of care-related services. To contextualize these findings, 61% of US adults stated that making childcare more affordable was a very or extremely important priority in 2023.^56^ High costs of long-term care combined with limited long-term care insurance coverage render older adults and their families few options for affordable care. Care from unpaid family or friends is the dominant source of care, despite more than half reporting feeling little to no choice in assuming this responsibility.^61,62,63^ Future research may consider changing patterns in national support of caregiver policies given changing political landscapes, public conversations on the importance of family, and the realities of an aging population. Future work may also consider the many assets that support family caregivers and changes in economic characteristics (eg, medical debt) and measures of well-being in caregivers of adults.^64^ Overall, study results highlight the desire across political parties for a national dialogue on access to affordable long-term care.

While reform of the long-term care system has been a priority of policymakers in recent decades, proposed reforms have either failed or ultimately been scaled back, falling short of ensuring dignified, appropriate, and affordable care for persons living with disability.^4,65,66,67,68^Reasons for these failures have included conflicts regarding whether care should be provided through public vs private insurance systems, as highlighted through the failure of the CLASS Act in 2011 and the Build Back Better Act in 2021.^69,70,71,72^ Similarly, while the 2022 National Strategy to Support Family Caregivers has gained momentum, financial support for overseeing the implementation of the strategy by the Administration for Community Living has been modest. For decades, recommendations from commissions representing the consensus from the scientific community and Congress have emphasized the human toll associated with inadequacies and fragmentation of long-term care.^1,12,67,73,74,75^ Most recently, the April 2022 release of the consensus study from National Academies of Sciences, Engineering, and Medicine called “The National Imperative to Improve Nursing Home Quality” called for reform of long-term care financing in the US.^67^ The current study’s results are noteworthy in suggesting that more incremental efforts to address affordability of care, supporting family caregivers, and investing in paid care workers have high bipartisan public support in 2024. While we found that political affiliation was a strong determinant of support for these policies, with Democrats more likely to support policies, we found that support among Republicans remained strong for most policies, particularly those to retain assets while accessing long-term care coverage and address the affordability of long-term care in facilities. Moreover, policies that allowed individuals to retain assets while accessing long-term care coverage had high levels of bipartisan support, and the greatest consensus across political affiliations was evidenced by the difference in the level of support by affiliation. Considering the overall level of support and consensus across political affiliations may further aid policy makers in prioritizing which initiatives to pursue. With renewed focus on the failings of the financing and delivery of long-term care system in the US in the wake of the COVID-19 pandemic combined with high levels of support,^3,67,76^ a policy window may be opening in coming years that may lead to substantial change in how long-term care is financed and provided.^44,77^

### Limitations

This study had several limitations. First, we did not measure political leaning, which may inform policy preferences more than political party affiliation. Relatedly, the sample may have underrepresented respondents who self-reported as Republicans (32% of voters but only 25% of the survey respondents in our analytic sample).^42^ Second, participants were asked to indicate support for each policy preference but were not asked how their support would change based on how each policy was funded. Third, we were unable to disentangle how support for these policies have changed over time due to the cross-sectional nature of the data and lack of available longitudinal data measuring support for long-term care policies, to the best of our knowledge. Finally, we did not capture the length of time that participants served as a caregiver or number of care recipients, given the possibility of overlapping caregiving episodes. Future work could explore these policy questions to better understand how policy support can translate to policy action.

## Conclusions

This cohort study found that political affiliation had a greater association with policies to support care for adults with disabilities and their families than sex, race, or caregiving experience. However, significant differences by sex within political affiliation exist, which may reflect the differential burden of caregiving by sex. With a politically divided populace, support from both major political parties will be necessary to pass legislation to address the care needs of persons living with disabilities and their families.

Enhancing the infrastructure to support care workers can help to address the growing needs of an aging population. Building on the bipartisan 2022 National Strategy to Support Family Caregivers, we find that there is widespread endorsement by US adults for policies that support family caregivers, with the greatest enthusiasm across political parties for policies that make long-term care in the home and facilities more affordable, allow adults to retain assets to receive long-term care services, and strengthen the paid caregiving workforce, suggesting that these are viable policies with high support across party lines.

## Notes

Supplement 1. eMethods. eTable 1. Unadjusted Percent Endorsing Policies by Caregiver Status and Political Affiliation eTable 2. Full Model Results eTable 3. Differential Association of Gender within Political Affiliation for Endorsement of Policies

Supplement 2. Data sharing statement
