# Supplementary material for: Support for Care Economy Policies by Political Affiliation and Caregiving Responsibilities
Source: JAMA Health Forum. 2025 Jun 6;6(6):e251204. doi: 10.1001/jamahealthforum.2025.1204 (PMC12144621; doi:10.1001/jamahealthforum.2025.1204)
Supplement: Supplement 1. — eMethods. eTable 1. Unadjusted Percent Endorsing Policies by Caregiver Status and Political Affiliation eTable 2. Full Model Results eTable 3. Differential Association of Gender within Political Affiliation for Endorsement of Policies [file jamahealthforum-e251204-s001.pdf]

Supplemental Online Content

Miller KEM, Wolff JL, Shen K, Galea S, Ettman CK. Political affiliation, caregiving responsibilities, and support for care economy policies. *JAMA Health Forum*. 2025;6(6):e251204. doi:10.1001/jamahealthforum.2025.1204

eMethods.

eTable 1. Unadjusted Percent Endorsing Policies by Caregiver Status and Political Affiliation

eTable 2. Full Model Results

eTable 3. Differential Association of Gender within Political Affiliation for Endorsement of Policies

This supplemental material has been provided by the authors to give readers additional information about their work.

eMethods.

Selected CLIMB Study Questions

1. Defining Family Caregivers

At any time in the last 12 months, has anyone in your household provided unpaid care to a relative or friend 18 years or older to help them take care of themselves?

This may include helping with personal needs or household chores. It might be managing a person's finances, arranging for outside services, or visiting regularly to see how they are doing. This adult does not need to live with you.

RESPONSE OPTIONS:

1. Yes, I have provided care to an adult in the last year
2. Someone else in my household has provided care (not me)
3. No

2. Defining Political Affiliation

Do you consider yourself a Democrat, a Republican, an Independent or none of these?

RESPONSE OPTIONS:

1. Democrat
2. Republican
3. Independent
4. None of these

3. Endorsement of Policies Supporting Adults with Disabilities and their families.

The federal government is considering a plan for family and other unpaid caregivers who help children and adults for health and functioning needs. Some of the policies that have been

© 2025 Miller KEM et al. *JAMA Health Forum*.

proposed are listed below. They would each cost money. On a scale of 1 to 5 where 1 is strongly oppose and 5 is strongly support, how much do you support each policy?

GRID ITEMS:

A. Provide paid family and medical leave (e.g., short term leave with partial pay)

B. Provide payments for family members or friends giving care

C. Make aging at home more affordable, through strengthening accessibility and quality of services

and supports (e.g., respite care, adult day care)

D. Make long term care at a facility (e.g., nursing home) more affordable

E. Do not require people to deplete their assets to be eligible for long-term care

F. Support and expand the professional caregiving workforce

RESPONSE OPTIONS:

01 Strongly Oppose

02 Somewhat Oppose

03 Neither Support nor Oppose

04 Somewhat Support

05 Strongly Support

© 2025 Miller KEM et al. *JAMA Health Forum*.

eTable1. Unadjusted Percent Endorsing Policies by Caregiver Status and Political Affiliation

	Not a Caregiver	Caregiver	Total
**Provide paid family and medical leave.**			
Democrat	0.8221 (0.0178)	0.7789 (0.0425)	0.8140 (0.0149)
Republican	0.4854 (0.0295)	0.4733 (0.0588)	0.4829 (0.0258)
Independent	0.6312 (0.0256)	0.6010 (0.0672)	0.6252 (0.0276)
None of these	0.6191 (0.0356)	0.6006 (0.0866)	0.6144 (0.0319)
**Provide payments for family members or friends giving care.**			
Democrat	0.7847 (0.0200)	0.7568 (0.0396)	0.7796 (0.0171)
Republican	0.4239 (0.0293)	0.5579 (0.0604)	0.4517 (0.0242)
Independent	0.5530 (0.0241)	0.6512 (0.0572)	0.5724 (0.0214)
None of these	0.5190 (0.0458)	0.7128 (0.0813)	0.5597 (0.0408)
**Make aging at home more affordable, through strengthening accessibility and quality of services and supports.**			
Democrat	0.8782 (0.0163)	0.8546 (0.0397)	0.8739 (0.0131)
Republican	0.6370 (0.0264)	0.7561 (0.0467)	0.6617 (0.0220)
Independent	0.7650 (0.0247)	0.7488 (0.0628)	0.7618 (0.0238)
None of these	0.6024 (0.0494)	0.6445 (0.0921)	0.6113 (0.0429)
**Make long term care at a facility more affordable.**			
Democrat	0.9003 (0.0162)	0.8628 (0.0362)	0.8934 (0.0146)
Republican	0.6906 (0.0315)	0.7137 (0.0612)	0.6954 (0.0257)
Independent	0.7961 (0.0275)	0.7724 (0.0602)	0.7914 (0.0260)
None of these	0.7088 (0.0429)	0.6442 (0.0884)	0.6952 (0.0379)
**Do not require people to deplete their assets to be eligible for long-term care.**			
Democrat	0.8213 (0.0191)	0.7896 (0.0418)	0.8154 (0.0179)
Republican	0.7006 (0.0270)	0.7550 (0.0624)	0.7119 (0.0266)
Independent	0.8111 (0.0240)	0.7662 (0.0520)	0.8022 (0.0203)
None of these	0.7263 (0.0333)	0.6961 (0.0904)	0.7200 (0.0307)
**Support and Expand the Paid Care Workforce**			
Democrat	0.8940 (0.0154)	0.8580 (0.0437)	0.8873 (0.0117)
Republican	0.6585 (0.0230)	0.7894 (0.0489)	0.6857 (0.0207)
Independent	0.7817 (0.0259)	0.8357 (0.0391)	0.7924 (0.0230)
None of these	0.6538 (0.0454)	0.7767 (0.0663)	0.6796 (0.0376)

© 2025 Miller KEM et al. *JAMA Health Forum*.

eTable 2. Full Model Results

	Marginal Effect (Standard Error)					
	Make Care in Facilities More Affordable	Support and Expand the Paid Care Workforce	Retain Assets to be eligible for long-term care coverage	Make Care at Home More Affordable	Paid Leave	Pay Family Caregivers
Respondent age	0.0026**	0.0027**	0.0025**	0.0031**	-0.0029**	-0.0018
	-0.001	-0.0009	-0.0009	-0.001	-0.0009	-0.001
White, non-Hispanic	-ref-	-ref-	-ref-	-ref-	-ref-	-ref-
Black, non-Hispanic	-0.1401**	-0.1328**	-0.1212**	-0.1338**	-0.0533	0.0224
	0.0469	0.0456	0.0440	0.0435	-0.0517	-0.0438
Hispanic	-0.0023	-0.0633	-0.0283	-0.0037	0.0439	0.0385
	0.0351	0.0373	0.0354	0.0359	0.0318	0.0373
All Other Races	-0.0040	-0.0298	-0.0782	-0.0225	0.0608	0.0588
	0.0439	0.0441	0.0451	0.0457	0.0461	0.0456
Less than HS	-ref-	-ref-	-ref-	-ref-	-ref-	-ref-
HS graduate or equivalent	0.0288	0.0164	0.0910	-0.0029	-0.0342	-0.0635
	0.0500	0.0511	0.0742	0.0559	0.0613	0.0608
Some college/ associates degree	0.0559	0.0616	0.0899	0.0289	-0.0230	-0.0813
	0.0476	0.0482	0.0686	0.0580	0.0600	0.0598
Bachelor's degree	0.0414	0.0215	0.0400	0.0257	-0.0247	-0.1341*
	0.0508	0.0489	0.0706	0.0552	0.0579	0.0640
Post grad study/professional degree	0.0522	0.0883	0.0643	0.0929	-0.0241	-0.1606*
	0.0567	0.0562	0.0829	0.0605	0.0633	0.0648
Married	-ref-	-ref-	-ref-	-ref-	-ref-	-ref-

© 2025 Miller KEM et al. *JAMA Health Forum.*

Widowed	-0.0534	-0.0550	-0.1134	-0.0817	0.0503	-0.0518
	0.0687	0.0584	0.0654	0.0597	0.0558	0.0656
Divorced	0.0023	0.0101	-0.0159	-0.0023	-0.0228	-0.0025
	0.0286	0.0362	0.0305	0.0406	0.0336	0.0326
Never married	-0.0182	0.0299	-0.0445	-0.0128	-0.0733*	-0.0719
	0.0311	0.0343	0.0354	0.0322	0.0355	0.0367
9-level Household Income	0.0051	0.0084*	0.0074*	0.0039	0.0044	0.0017
	0.0031	0.0034	0.0033	0.0036	0.0033	0.0037
Household size (including children)	0.0089	0.0148*	0.0132*	0.0069	0.0083	0.0032
	0.0055	0.0060	0.0060	0.0064	0.0062	0.0070
Not a Parent	-ref-	-ref-	-ref-	-ref-	-ref-	-ref-
Parent	-0.0810***	-0.0200	-0.0571*	-0.0575*	-0.0361	-0.0324
	0.0240	0.0321	0.0253	0.0284	0.0303	0.0328
New England	-ref-	-ref-	-ref-	-ref-	-ref-	-ref-
Mid-Atlantic	-0.0323	-0.0136	0.0321	0.0453	-0.1321*	-0.0043
	0.0494	0.0614	0.0297	0.0821	0.0601	0.0609
East North Central	-0.0686	-0.0460	-0.0505	0.0435	-0.0737	-0.0095
	0.0463	0.0652	0.0415	0.0788	0.0511	0.0583
West North Central	-0.0584	-0.0177	-0.0518	0.0200	-0.0770	-0.0741
	0.0561	0.0716	0.0480	0.0902	0.0638	0.0695
South Atlantic	-0.0507	-0.0590	-0.0296	0.0048	-0.0691	-0.0386
	0.0456	0.0652	0.0394	0.0787	0.0491	0.0581
East South Central	-0.0370	0.0025	0.0305	0.0148	-0.1224*	0.0227
	0.0551	0.0740	0.0523	0.0818	0.0577	0.0804
West South Central	-0.0726	-0.0469	-0.0362	-0.0171	-0.1361*	-0.0715
	0.0671	0.0724	0.0550	0.0926	0.0651	0.0734
Mountain	-0.0756	-0.0037	-0.0196	0.0260	-0.0448	-0.0136
	0.0508	0.0765	0.0519	0.0817	0.0583	0.0692
Pacific	-0.0236	0.0165	-0.0244	0.0120	-0.0753	0.0021

© 2025 Miller KEM et al. *JAMA Health Forum.*

	0.0497	0.0641	0.0433	0.0821	0.0569	0.0673
Non-Metro Area	-ref-	-ref-	-ref-	-ref-	-ref-	-ref-
Metro Area	-0.0575*	-0.0179	-0.0120	-0.0212	-0.0216	-0.0089
	0.0268	0.0279	0.0312	0.0300	0.0410	0.0329
Male	-ref-	-ref-	-ref-	-ref-	-ref-	-ref-
Female	0.0414	0.0758***	0.0325	0.0817**	0.1076***	0.0874***
	0.0231	0.0213	0.0238	0.0248	0.0262	0.0233
Not a Caregiver	-ref-	-ref-	-ref-	-ref-	-ref-	-ref-
Caregiver	-0.0274	0.0485	-0.0207	0.0126	-0.0313	0.0702*
	0.0318	0.0297	0.0298	0.0325	0.0344	0.0322
Democrat	-ref-	-ref-	-ref-	-ref-	-ref-	-ref-
Republican	-0.2175***	-0.2173***	-0.1372***	-0.2224***	-0.3336***	-0.3286***
	0.0295	0.0240	0.0336	0.0269	0.0287	0.0287
Independent	-0.0971***	-0.0919***	-0.0129	-0.1002***	-0.1883***	-0.1930***
	0.0262	0.0243	0.0257	0.0234	0.0314	0.0273
None of these	-0.1443***	-0.1631***	-0.0525	-0.2052***	-0.2173***	-0.2418***
	0.0378	0.0334	0.0335	0.0432	0.0394	0.0443
N	2059	2059	2059	2059	2059	2059

* p<0.05, ** p<0.01, *** p<0.001

© 2025 Miller KEM et al. *JAMA Health Forum*.

eTable 3. Differential Association of Gender within Political Affiliation for Endorsement of Policies

	Marginal Effect (Standard Error)					
	Make Care in Facilities More Affordable	Support and Expand the Paid Care Workforce	Retain Assets to be eligible for long-term care coverage	Make Care at Home More Affordable	Paid Leave	Pay Family Caregivers
Democrats						
Male	-ref-	-ref-	-ref-	-ref-	-ref-	-ref-
Female	-0.0339	-0.0183	-0.0130	-0.0295	-0.0250	-0.0268
	(0.0317)	(0.0381)	(0.0388)	(0.0342)	(0.0376)	(0.0367)
Republicans						
Male	-ref-	-ref-	-ref-	-ref-	-ref-	-ref-
Female	0.0649	0.0718	0.0765	0.0932	0.1862***	0.1577**
	(0.0591)	(0.0565)	(0.0505)	(0.0594)	(0.0556)	(0.0567)
Independents						
Male	-ref-	-ref-	-ref-	-ref-	-ref-	-ref-
Female	0.0967**	0.1516***	0.0416	0.1636***	0.1610***	0.0973
	(0.0342)	(0.0419)	(0.0358)	(0.0352)	(0.0484)	(0.0517)
None of these						
Male	-ref-	-ref-	-ref-	-ref-	-ref-	-ref-
Female	0.0788	0.1672*	0.0525	0.1793	0.2009**	0.2349***
	(0.0678)	(0.0734)	(0.0674)	(0.0983)	(0.0770)	(0.0701)
N	2059	2059	2059	2059	2059	2059

* p<0.05, ** p<0.01, *** p<0.001

© 2025 Miller KEM et al. *JAMA Health Forum*.
