# Supplementary material for: Support for Care Economy Policies by Political Affiliation and Caregiving Responsibilities
Source: JAMA Health Forum. 2025 Jun 6;6(6):e251204. doi: 10.1001/jamahealthforum.2025.1204 (PMC12144621; doi:10.1001/jamahealthforum.2025.1204)
Supplement: Supplement 2. — Data sharing statement [file jamahealthforum-e251204-s002.pdf]

Data Sharing Statement

Miller. Support for Care Economy Policies by Political Affiliation and Caregiving Responsibilities. *JAMA Health Forum*. Published June 06, 2025.
doi:10.1001/jamahealthforum.2025.1204

Data
Data available: Yes
Data types: Deidentified participant data
How to access data: Deidentified participant data may be available upon request. Please contact the corresponding author at kmill177@jh.edu
When available: With publication

Supporting Documents
Document types: Statistical/analytic code
How to access documents: Statistical/analytic code will be made available upon request. Please contact the corresponding author at kmill177@jh.edu
When available: With publication

Additional Information
Who can access the data: Data are available upon reasonable request for research purposes to the CLIMB team.
Types of analyses: Data will be made available for a specified purpose.
Mechanisms of data availability: Data will be made available with a signed data access agreement.
